# Diagnostic and prognostic performance of the ratio between high-sensitivity cardiac troponin I and troponin T in patients with chest pain

**DOI:** 10.1371/journal.pone.0276645

**Published:** 2022-11-01

**Authors:** Kai M. Eggers, Ola Hammarsten, Sally J. Aldous, Louise Cullen, Jaimi H. Greenslade, Bertil Lindahl, William A. Parsonage, Christopher J. Pemberton, John W. Pickering, A. Mark Richards, Richard W. Troughton, Martin P. Than

**Affiliations:** 1 Department of Medical Sciences and Uppsala Clinical Research Center, Uppsala University, Uppsala, Sweden; 2 Department of Clinical Chemistry, Sahlgrenska University Hopsital, Göteborg, Sweden; 3 Department of Cardiology, Christchurch Hospital, Christchurch, New Zealand; 4 Emergency Department, Royal Brisbane and Women’s Hospital, Brisbane, Australia; 5 Department of Cardiology, Royal Brisbane and Women’s Hospital, Brisbane, Australia; 6 Christchurch Heart Institute, Department of Medicine, University of Ontago, Christchurch, New Zealand; 7 Emergency Department, Christchurch Hospital, Christchurch, New Zealand; 8 Cardiovascular Research Institute, National University of Singapore, Singapore, Singapore; University of California, Davis, UNITED STATES

## Abstract

**Background:**

Elevations of high-sensitivity cardiac troponin (hs-cTn) concentrations not related to type 1 myocardial infarction are common in chest pain patients presenting to emergency departments. The discrimination of these patients from those with type 1 myocardial infarction (MI) is challenging and resource-consuming. We aimed to investigate whether the hs-cTn I/T ratio might provide diagnostic and prognostic increment in this context.

**Methods:**

We calculated the hs-cTn I/T ratio in 888 chest pain patients having hs-cTnI (Abbott Laboratories) or hs-cTnT (Roche Diagnostics) concentrations above the respective 99^th^ percentile at 2 hours from presentation. All patients were followed for one year regarding mortality.

**Results:**

The median hs-cTn I/T ratio was 3.45 (25^th^, 75^th^ percentiles 1.80–6.59) in type 1 MI patients (n = 408 ☯46.0%]), 1.18 (0.81–1.90) in type 2 MI patients (n = 56 ☯6.3%]) and 0.67 (0.39–1.12) in patients without MI. The hs-cTn I/T ratio provided good discrimination of type 1 MI from no type 1 MI (area under the receiver-operator characteristic curve 0.89 ☯95% confidence interval 0.86–0.91]), of type 1 MI from type 2 MI (area under the curve 0.81 ☯95% confidence interval 0.74–0.87]), and was associated with type 1 MI in adjusted analyses. The hs-cTn I/T ratio provided no consistent prognostic value.

**Conclusions:**

The hs-cTn I/T ratio appears to be useful for early diagnosis of type 1 MI and its discrimination from type 2 MI in chest pain patients presenting with elevated hs-cTn. Differences in hs-cTn I/T ratio values may reflect variations in hs-cTn release mechanisms in response to different types of myocardial injury.

## Introduction

Acute chest pain is one of the most common presentations in the Emergency Department (ED). The differential diagnosis ranges from non-serious (e.g. musculoskeletal problems, anxiety) to potentially life-threatening conditions (e.g. acute myocardial necrosis due to type 1 myocardial infarction ☯MI]). Measurement of cardiac troponin (cTn) concentrations is a key component in the assessment of this heterogeneous population. Early assessment has been improved by the implementation of high-sensitivity (hs) assays, in particular with respect to the exclusion of MI. However, the clinical implementation of hs-assays has also been associated with increased numbers of patients with elevated cTn concentrations for whom the underlying cause is not immediately clear. This includes patients with non-ischemic myocardial injury or type 2 MI reflecting myocardial oxygen supply/demand mismatch which in most cases is due to non-coronary conditions. Chronic hs-cTn elevation can also be caused by non-necrotic mechanisms, e.g. cardiomyocyte apoptosis or decreased renal clearance [1], and this can be difficult to distinguish from the hs-cTn pattern observed in late-presenting patients with type 1 MI.

Using current assessment approaches, the differential diagnosis of the cause of cTn release can be challenging and must often be based on clinical, laboratory and imaging results obtained during the hospitalization. However, the distinction of type 1 MI from non-coronary causes of cTn elevation apparent already upon ED presentation is important. This would allow for immediate allocation of appropriate management while avoiding overuse of hospital resources and deployment of potentially harmful interventions.

The cTn I/T ratio could be helpful in addressing these challenges. Although cTnT and cTnI are expressed as an obligate 1:1 complex in cardiac tissue [2] and cleared with the same kinetics once they reach circulation [3], peak cTnI concentrations are often ten-times higher in type 1 MI patients compared to cTnT [4–6]. Higher concentrations of cTnT in contrast, are often observed in situations without obvious cardiomyocyte necrosis, e.g. limited cardiac ischemia [7, 8], stable atrial fibrillation [9], chronic kidney disease [10, 11] and also in the general population [12]. Accordingly, the relative concentrations of cTnT and cTnI might provide valuable information for the early work-up of patients with cardiac complaints. The aims of the present study were thus, to investigate whether the hs-cTn I/T ratio might distinguish type 1 MI from no type 1 MI, and type 1 MI from type 2 MI in chest pain patients presenting with elevated hs-cTn concentrations.

## Material and methods

### Study population

We used data collected from four studies (five cohorts) of chest pain patients presenting between 2007 and 2018 to ED’s in Brisbane, Australia and Christchurch, New Zealand. These studies were (i) the Accelerated Diagnostic Protocol to Assess patients with chest Pain symptoms using contemporary Troponins as the only biomarker (ADAPT) [13] and (ii) the Signal Peptides in Acute Coronary Events (SPACE) observational studies [14, 15], and (iii) the ADAPT-Accelerated Diagnostic Pathway (ADAPT-ADP) [16] and (iv) the Emergency Department Assessment of Chest pain Score (EDACS) [17] randomized controlled trials. All studies were carried out according to the principles of the Declaration of Helsinki and were approved by local ethics committees (ADAPT, ADAPT-RCT: Upper South A Regional Ethics Committee, Christchurch, New Zealand; SPACE, EDACS: Central Regional Health and Disability Ethics Committee, Wellington, New Zealand). Written informed consent was obtained from all patients. All studies were registered at the Australia-New Zealand Clinical Trials Registry (ADAPT: ACTRN12611001069943, SPACE: ACTRN12611001076965, ADAPT-RCT: ACTRN12610000766011, EDACS: ACTRN12613000745741).

The studies employed very similar inclusion and exclusion criteria. Eligible patients were aged ≥18 years and presented acutely from the community to the ED with chest pain suggestive of MI. Patients were excluded if any of the following conditions were present: ST elevation MI on any ECG, onset of chest pain >12 h prior to assessment, proven or suspected non-coronary pathology as cause of chest pain, need for admission due to other medical conditions or need for other investigations, previous study enrolment, anticipated problems with follow-up (e.g. resident outside the country or terminal illness), and inability or unwillingness to provide informed consent [14–17]. All patients underwent routine clinical assessment which was left to the discretion of the attending physicians.

The merged database from the four study cohorts consisted of 3790 patients. A total of 2870 patients had results for hs-cTnI and hs-cTnT available from samples obtained at presentation, and 3124 patients had 2-hour results for both hs-cTnI and hs-cTnT. Given the explorative character of our analysis and in order to improve statistical power, we focused on 2-hour hs-cTn results but considered results obtained at ED presentation together with 2-hour results in secondary analyses of change in hs-cTn I/T ratio. Because of our focus on the differential diagnosis of hs-cTn elevation, we did not consider patients with 2-hour hs-cTnI or hs-cTnT concentrations at or below the respective 99^th^ percentile. Patients with an adjudicated diagnosis of ST-elevation MI were excluded.

### Diagnostic classification

The index diagnoses were independently adjudicated in all studies by clinicians not involved in the management of the patients, see S1 Appendix for details. The diagnosis of MI was based on criteria outlined in the Universal Definition [18, 19], requiring evidence of a rise or fall in cTn concentrations measured at presentation and after≥6 hours with at least one concentration above the 99^th^ percentile together with clinical or electrocardiographic evidence of myocardial ischemia. The reference (local laboratory) cTn assays in use for the adjudication of MI for each cohort were: Access AccuTnI (Beckman Coulter, Chaska, MN) for the ADAPT Brisbane cohort, Architect cTnI (Abbot Diagnostics, Chicago, IL) for the ADAPT Christchurch cohort and ADAPT-ADP, and Architect hs-cTnI (Abbot Diagnostics) for SPACE and EDACS. A significant cTn rise or fall was normally defined as a 20% change. For the Christchurch cohorts, a change was not rigidly specified although 20% was commonly used. Type 2 MI was defined as MI that met Universal Definition requirements, but where a condition other than coronary thrombosis contributed to an imbalance between myocardial oxygen supply/demand [18, 19].

### Hs-cTn sampling and analysis

In all studies, samples for analysis of hs-cTn were obtained at prespecified timepoints [13, 15–17]. After blood draw, samples were stored frozen in aliquots at -80° until analysis. cTn was measured using the Abbott hs-cTnI and the Roche hs-cTnT (Roche Diagnostics, Basel, Switzerland) assays. The level of detection of the hs-cTnI assay is <2 ng/L [20]. According to the manufacturer, the sex-specific 99^th^ percentiles derived from a healthy population are 16 ng/L (women) and 34 ng/L (men) with an overall 99^th^ percentile of 26 ng/L. The level of detection of the hs-cTnT assay is 5 ng/L, and the overall 99^th^ percentile is 14 ng/L [21]. The lowest concentration assuring a 10% coefficient of variation is below the respective 99^th^ percentile for both assays [20, 21]. The hs-cTnT results provided in the dataset had been appropriately corrected for the miscalibration affecting hs-cTnT lots used in 2010–2012 [22].

### Prognostic evaluation

The outcomes investigated in our analysis were all-cause mortality, cardiovascular and non-cardiovascular mortality within one year from ED presentation. Information on mortality had been obtained from national health registries.

### Statistical analysis

The hs-cTn I/T ratio was calculated from raw data. The discriminative value of the hs-cTn I/T ratio with respect to type 1 MI vs no type 1 MI and type 1 MI vs type 2 MI was assessed by calculation of the area under receiver-operator characteristic curves (AUC).

To investigate whether the predictive value of the hs-cTn I/T ratio regarding the presence of type 1 MI might be modified by clinical variables or confounders, multiple linear regressions were performed. Adjustment was made for year of study inclusion, study cohort, age, sex, time from onset of symptoms to presentation, previous smoking, hypertension, diabetes, hyperlipidemia, estimated glomerular filtration rate (CKD-EPI equation), previous MI, previous coronary revascularization, heart failure, previous stroke, peripheral artery disease and either hs-cTnI (model 1) or hs-cTnT (model 2). Concentrations of hs-cTnI and hs-cTnT, and hs-cTn I/T ratio values were ln-transformed before being entered into the analyses due to right skew.

The association of the hs-cTn I/T ratio with 1-year mortality was investigated using multivariable logistic regressions. Due to partly small event numbers, a limited adjustment set was applied using age, sex, year of study inclusion, study cohort, time from onset of symptoms to presentation and either hs-cTnI (model 1) or hs-cTnT (model 2) as covariates.

Continuous variables are reported as medians with 25^th^ and 75^th^ percentiles with comparisons made using the Mann-Whitney U-test. Categorical variables are expressed as frequencies and percentages. The software packages SPSS 27.0 (SPSS Inc., Chicago, IL) and Stata 17 (Stata Corp., College Station, TX) were used for the analyses.

## Results

**Fig 1:**
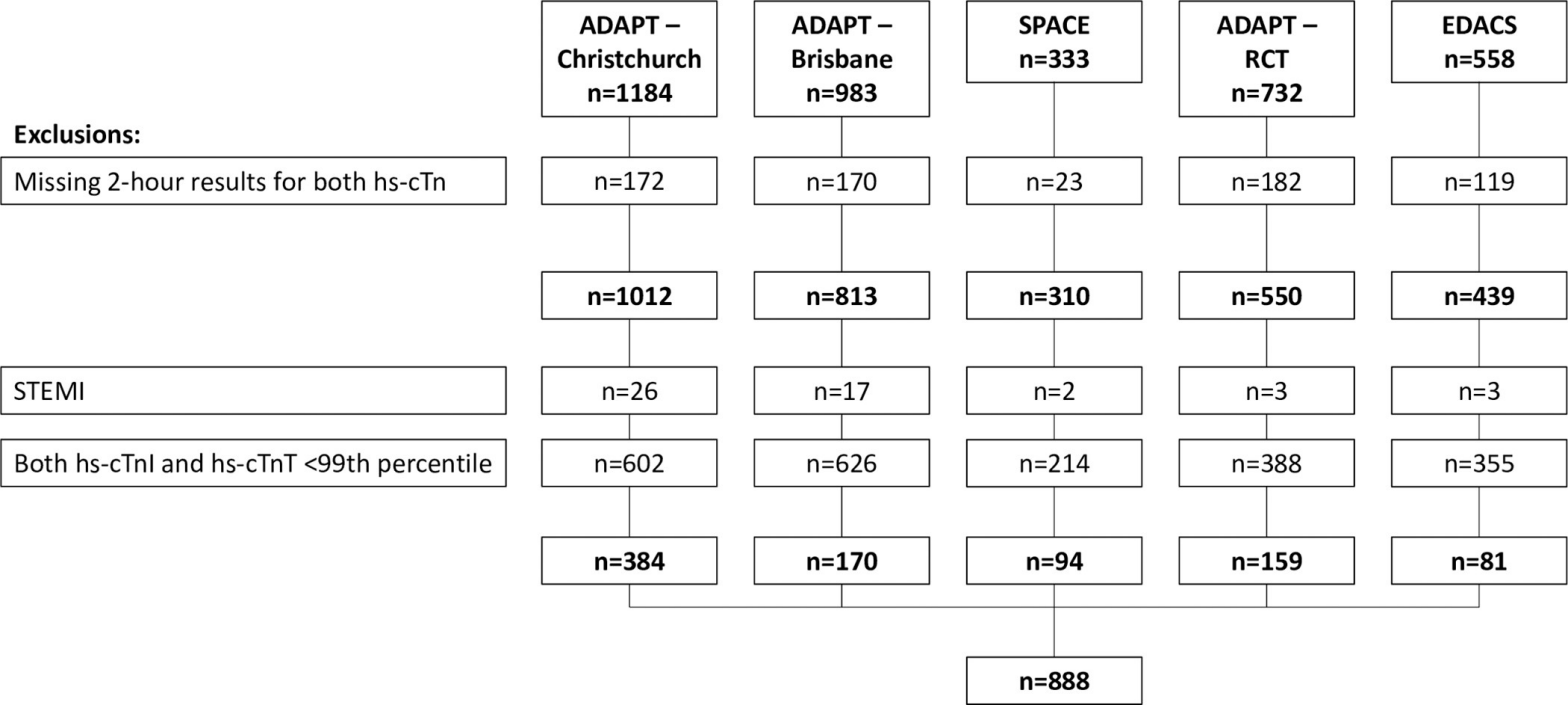
Study flowchart. STEMI: ST-elevation myocardial infarction.

Two-hour results for hs-cTnI and hs-cTnT were available in 3124 patients. Following exclusion of 51 patients with an adjudicated diagnosis of ST-elevation MI and of 2185 patients with hs-cTnI or hs-cTnT at or below both the respective 99^th^ percentiles, 888 patients were included in subsequent analyses (Fig 1). Four-hundred eight (46.0%) patients had type 1 MI and 56 (6.3%) patients had type 2 MI. The remaining 424 (47.7%) patients were regarded as having myocardial injury due to other mechanisms.

**Fig 2:**
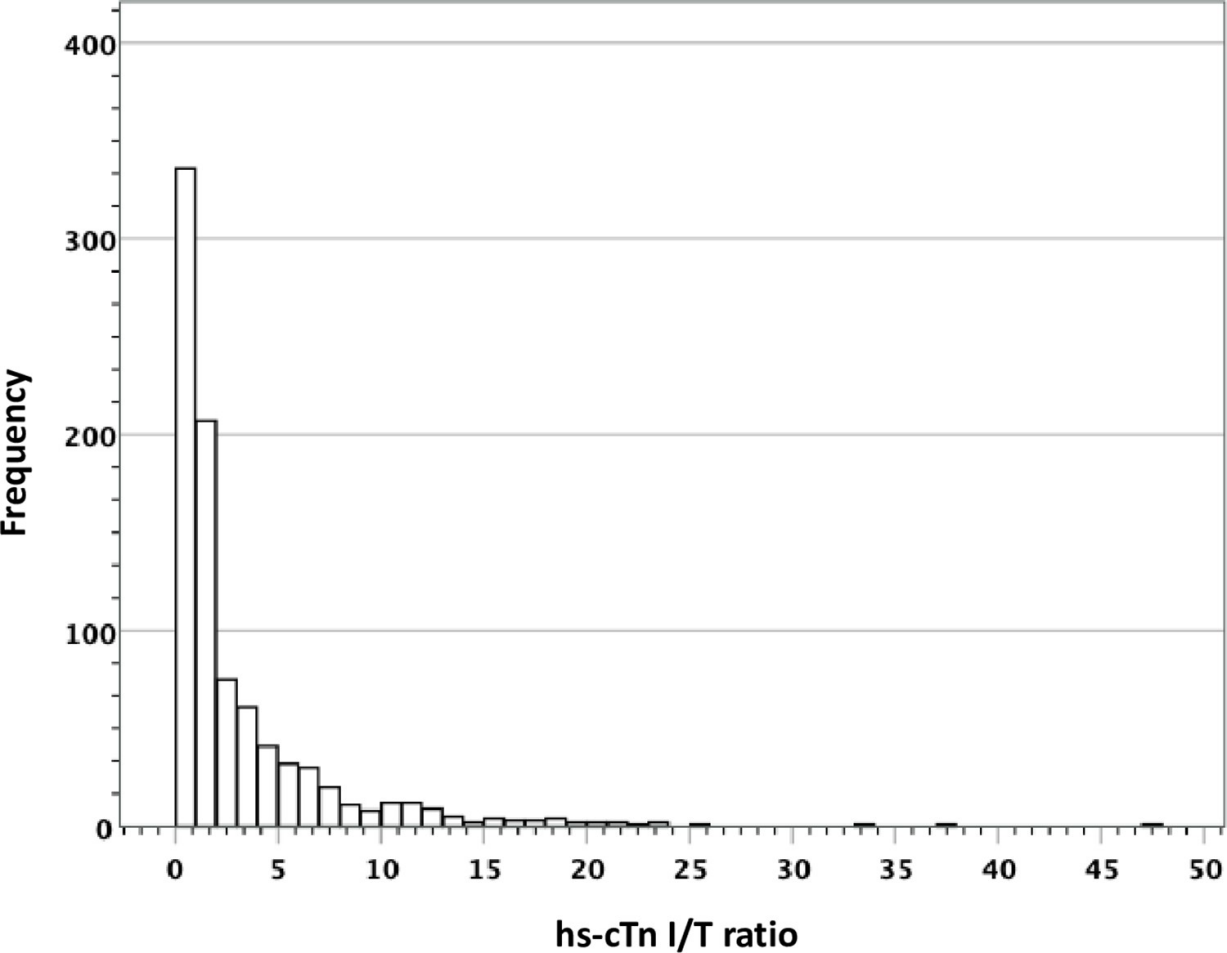
Distribution of hs-cTn I/T ratio values.

Table 1 presents information on clinical characteristics, hs-cTn results and 1-year mortality. Corresponding data per study cohort is given in S1 Table. The correlation of hs-cTnI and hs-cTnT was high (Spearman‘s rank correlation: r = 0.78; p<0.001). Five-hundred four (56.8%) patients had hs-cTn concentrations above the 99^th^ percentiles for both assays, 33 (3.7%) patients had isolated hs-cTnI elevation and 351 (39.5%) patients had isolated hs-cTnT elevation. For the total study population, the hs-cTn I/T ratio was 1.41 (25^th^, 75^th^ percentiles 0.65–3.85), see Fig 2. The hs-cTn I/T ratios differed considerably among the three diagnostic cohorts with highest values in type 1 MI (3.45 [1.80–6.59]), followed by type 2 MI (1.18 [0.81–1.90]) and myocardial injury (0.67 [0.39–1.12]). S1 and S2 Figs depict the distribution of hs-cTnI and hs-cTnT concentrations in relation to the presence of type 1 MI.

**Table 1:** Clinical characteristics and 1-year outcome.

	Type 1 MI (n = 408)	Type 2 MI (n = 56)	Myocardial injury (n = 424)	Total (n = 888)
**Demographics**				
Age (years)	69.6 (59.3–78.6)	72.5 (63.3–80.0)	72.8 (63.6–80.2)	71.0 (61.0–79.8)
Men	295 (72.3%)	26 (46.4%)	286 (67.5%)	607 (68.4%)
**Risk factors**				
Previous smoking	72 (17.6%)	8 (14.3%)	51 (12.0%)	131 (14.8%)
Hypertension	253 (62.0%)	39 (69.6%)	303 (71.6%)	595 (67.1%)
Diabetes	84 (20.6%)	14 (25.0%)	99 (23.3%)	197 (22.2%)
Hyperlipidemia	236 (57.8%)	30 (53.6%)	286 (67.6%)	552 (62.2%)
Body mass index (kg/m^2^)*	27.8 (24.9–31.5)	26.2 (21.9–29.9)	27.6 (24.3–31.4)	27.6 (24.6–31.4)
eGFR (mL/min/1.73m^2^)†	67.3 (53.6–80.5)	58.2 (38.6–75.3)	61.9 (46.0–76.9)	64.6 (48.5–78.8)
**Comorbidities**				
Previous MI	142 (34.8%)	20 (35.7%)	186 (43.9%)	348 (39.2%)
Previous PCI	105 (25.7%)	14 (25.0%)	133 (31.4%)	252 (28.4%)
Previous CABG	48 (11.8%)	6 (10.7%)	73 (17.2%)	127 (14.3%)
Heart failure	32 (7.8%)	7 (12.5%)	79 (18.6%)	118 (13.3%)
Previous stroke	32 (7.8%)	11 (19.6%)	62 (14.6%)	105 (11.8%)
Peripheral artery disease	33 (8.1%)	3 (5.4%)	35 (8.3%)	71 (8.0%)
**Time from onset of symptoms (hours)** ‡	5.9 (2.2–12.8)	5.6 (1.6–32.4)	4.1 (2.1–9.3)	4.7 (2.1–12.0)
**Ischemic ECG**	112 (27.5%)	15 (26.8%)	49 (11.6%)	176 (19.8%)
**hs-cTn results (2 hours)**				
hs-cTnI (ng/L)	232 (75–1170)	44 (28–93)	15 (8–26)	41 (15–238)
hs-cTnT (ng/L)	75 (38–217)	36 (26–63)	21 (16–30)	32 (19–80)
hs-cTn I/T ratio	3.45 (1.80–6.59)	1.18 (0.81–1.90)	0.67 (0.39–1.12)	1.41 (0.65–3.85)
**1-year outcome** §				
All-cause mortality	35 (8.8%)	8 (16.7%)	26 (6.7%)	69 (8.3%)
• CV mortality	30 (7.6%)	5 (10.4%)	10 (2.6%)	45 (5.4%)
• Non-CV mortality	5 (1.3%)	3 (6.3%)	16 (4.1%)	24 (2.9%)

*n = 821

†n = 724

‡n = 869

§ n = 834.

eGFR: estimated glomerular filtration rate; MI: myocardial infarction; PCI: percutaneous coronary intervention; CABG: coronary artery bypass grafting; cTn: cardiac troponin; CV: cardiovascular.

**Fig 3:**
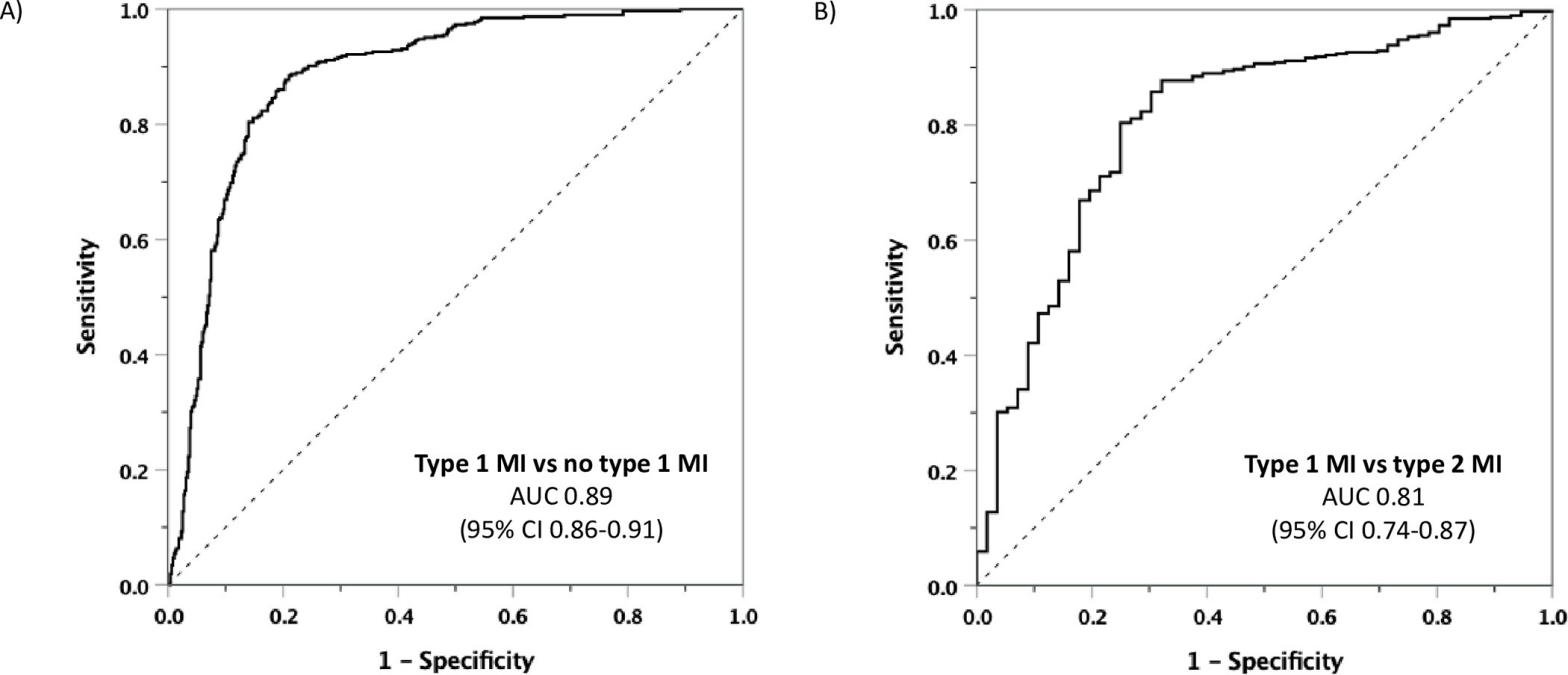
Discriminative value of the hs-cTn I/T ratio. A) Type 1 MI vs no type 1 MI; B) Type 1 MI vs type 2 MI. MI: myocardial infarction; AUC: area under the receiver-operator characteristic curve; CI: confidence interval.

The hs-cTn I/T ratio provided high discriminative value regarding type 1 MI vs no type 1 MI with an AUC of 0.89 (95% confidence interval [CI] 0.86–0.91; Fig 3A). A hs-cTn I/T ratio of 1.00 had a specificity of 63.7 (95% CI 59.3–68.1)% for type 1 MI with a positive predictive value of 68.5 (95% CI 64.4–72.3)%. Specificities of 80% and 90% were achieved by hs-cTn I/T ratios of 1.40 and 2.24, respectively with corresponding positive predictive values of 78.5 (95% CI 74.4–82.2)% and 85.0 (95% CI 80.7–88.8)%. The AUC of the hs-cTn I/T ratio was higher compared to the AUC of hs-cTnT but smaller compared to the AUC of hs-cTnI (Table 2).

**Table 2:** Discriminative value of the hs-cTn I/T ratio compared to hs-cTnI or hs-cTnT.

	Type 1 MI vs no type 1 MI		Type 1 MI vs type 2 MI	
	AUC (95% CI)	p-value	AUC (95% CI)	p-value
Hs-cTn I/T	0.89 (0.86–0.91)	-	0.81 (0.74–0.87)	-
Hs-cTnI	0.91 (0.90–0.93)	<0.001	0.79 (0.73–0.85)	0.333
Hs-cTnT	0.84 (0.81–0.86)	0.001	0.69 (0.63–0.76)	0.002

P-values refer to comparisons with the AUC of the hs-cTn I/T ratio.

MI: myocardial infarction; AUC: area under the receiver-operator characteristic curve; CI: confidence interval.

For the discrimination of type 1 MI from type 2 MI, the AUC of the hs-cTn I/T ratio was 0.81 (95% CI 0.74–0.87), see Fig 3B. Hs-cTn I/T ratios of 2.17 and 4.14 provided specificities of 80% and 90%, respectively, with corresponding positive predictive values of 96.2 (95% CI 93.3–98.1)% and 96.6 (95% CI 92.8–98.8)%. The AUC of the hs-cTn I/T ratio was higher compared to the corresponding estimates for hs-cTnT and hs-cTnI, respectively (Table 2). Sensitivities and specificities across increasing hs-cTn I/T ratio values are presented in S3 Fig.

Results for the hs-cTn I/T ratio on presentation and at 2 hours were available in 783 patients. From presentation to 2 hours, the hs-cTn I/T ratio increased from 1.13 (0.57–2.95) to 1.39 (0.65–3.94) overall due to a more pronounced increase in hs-cTnI concentrations (from 30 [12–156] ng/L to 40 [15–258] ng/L) compared to hs-cTnT concentrations (increase from 29 [18–64] ng/L to 32 [19–85] ng/L), see S2 Table. The relative increase in the hs-cTn I/T ratio was numerically greater in patients with type 1 MI compared to those with type 2 MI (19.4 [3.2–55.0]% vs 10.5 [0.9–51.0]%; p = 0.343).

The presence of type 1 MI emerged as a strong predictor of a higher hs-cTn I/T ratio in models considering other clinical variables or confounders (Tables 3A and 3B). Hs-cTnI (ln) exhibited stronger associations with the hs-cTn I/T ratio compared to hs-cTnT (ln) with non-overlapping 95% CI for the regression coefficients in the overall cohort (B = 0.491 [95% CI 0.465–0.517] vs B = 0.344 [95% CI 0.275–0.412]). Even a higher estimated glomerular filtration rate exhibited consistent positive associations with the hs-cTn I/T ratio.

**Table 3:** Predictors of the hs-cTn I/T ratio (ln). A) Total cohort (n = 702); B) MI patients (n = 376).

**A)**	**Model 1**		**Model 2**	
	**β**	**p-value**	**β**	**p-value**
Age	-0.035	0.095	-0.057	0.098
Men	-0.017	0.322	-0.042	0.136
Previous smoking	-0.015	0.403	-0.016	0.573
Hypertension	0.008	0.667	-0.011	0.723
Diabetes	-0.007	0.668	-0.032	0.262
Hyperlipidemia	0.002	0.929	-0.046	0.131
eGFR	0.089	<0.001	0.121	<0.001
Previous MI	0.028	0.199	0.023	0.511
Previous PCI/CABG	0.038	0.081	0.045	0.201
Heart failure	-0.026	0.152	-0.034	0.251
Previous stroke	-0.008	0.655	-0.011	0.689
Peripheral artery disease	-0.049	0.004	-0.061	0.028
hs-cTnI (ln)	0.842	<0.001	-	-
hs-cTnT (ln)	-	-	0.323	<0.001
Type 1 MI vs no type 1 MI	0.054	0.02	0.428	<0.001
R^2^-coefficient	0.814		0.509	
**B)**	**Model 1**		**Model 2**	
	**β**	**p-value**	**β**	**p-value**
Age	-0.039	0.269	-0.023	0.661
Men	-0.051	0.062	-0.08	0.051
Previous smoking	-0.039	0.162	-0.05	0.242
Hypertension	-0.017	0.557	-0.051	0.25
Diabetes	0.04	0.149	0.048	0.245
Hyperlipidemia	-0.054	0.066	-0.113	0.011
eGFR	0.164	<0.001	0.253	<0.001
Previous MI	0.046	0.182	0.053	0.312
Previous PCI/CABG	0.062	0.071	0.076	0.142
Heart failure	0.008	0.765	0	0.995
Previous stroke	0.01	0.703	0.017	0.668
Peripheral artery disease	-0.031	0.24	-0.027	0.507
hs-cTnI (ln)	0.803	<0.001	-	-
hs-cTnT (ln)	-	-	0.519	<0.001
Type 1 MI vs type 2 MI	0.101	<0.001	0.248	<0.001
R^2^-coefficient	0.77		0.472	

Models 1 and 2 were adjusted for all listed variables along with year of study inclusion, study cohort and delay from symptom onset to presentation

eGFR: estimated glomerular filtration rate; MI: myocardial infarction; PCI: percutaneous coronary intervention; CABG: coronary artery bypass grafting.

Eight-hundred thirty-four patients had consented to 1-year follow-up. Death occurred in 69 of these patients (8.3%) and was due to cardiovascular causes in 45 cases (Table 1; S1 Table). The hs-cTn I/T ratio provided no prognostic value apart for non-cardiovascular mortality with odds ratios below 1 in the overall cohort. However, 95% CI were wide due to the limited number of events (Table 4). The interactions of the presence of type 1 MI vs no type 1 MI, and of type 1 MI vs type 2 MI on the associations of the hs-cTn I/T ratio with 1-year all-cause mortality were non-significant (p _interaction_ 0.110 and 0.083, respectively).

**Table 4:** Prognostic evaluation—Association of the hs-cTn I/T ratio (ln) with 1-year mortality.

		Model 1		Model 2	
**Total cohort (n = 815)**	**N events**	**OR (95% CI)**	**p-value**	**OR (95% CI)**	**p-value**
All-cause mortality	69	0.63 (0.37–1.07)	0.089	0.86 (0.64–1.16)	0.323
CV mortality	45	0.89 (0.47–1.68)	0.711	1.13 (0.78–1.62)	0.521
Non-CV mortality	24	0.44 (0.19–1.00)	0.051	0.58 (0.37–0.90)	0.016
**MI patients (n = 438)**	**N events**	**OR (95% CI)**	**p**	**OR (95% CI)**	**p**
All-cause mortality	43	0.52 (0.23–1.16)	0.111	0.66 (0.40–1.08)	0.096

Due to the limited numbers of deaths among MI patients, CV mortality and non-CV mortality were not considered in this subgroup.

Model 1: adjusted age, sex, year of study inclusion, study cohort, delay from symptom onset to presentation and hs-cTnI (ln)

Model 2: adjusted age, sex, year of study inclusion, study cohort, delay from symptom onset to presentation and hs-cTnT (ln).

OR: odds ratio; CI: confidence interval; CV cardiovascular; MI: myocardial infarction.

## Discussion

We report for the first time that the hs-cTn I/T ratio may help to identify type 1 MI in chest pain patients with elevated hs-cTn concentrations. The hs-cTn I/T ratio offered good diagnostic discrimination with an AUC of 0.89. Moreover, the hs-cTn I/T ratio performed well regarding the discrimination of type 1 MI from type 2 MI with an AUC of 0.81. The hs-cTn I/T ratio was strongly associated with the presence of type 1 MI, even in the context of other clinical variables or confounders.

The implementation of hs-cTn assays and well-defined decision algorithms has improved assessment of chest pain patients, in particular with respect to the early exclusion of MI and CV risk (‘rule-out’). However, the early identification of MI (‘rule-in’) in patients presenting with elevated hs-cTn concentrations can sometimes still be challenging. This piece of diagnostic work-up is straightforward if there is a typical presentation and a significant hs-cTn change. Still, the distinction of type 2 MI from type 1 MI can be problematic, especially in patients with known coronary artery disease or critical illness [23]. Stable hs-cTn elevation is moreover, often seen in patients presenting late after a coronary event, and this may be difficult to distinguish from chronically elevated hs-cTn concentrations such as observed in elderly patients, those with cardiovascular comorbidities or kidney disease.

The results of our investigation demonstrate that the hs-cTn I/T ratio could be a useful tool to overcome some of these problems. Our findings appear to be explained by differences in the release mechanisms of cTnI and cTnT from necrotic myocardium. Similar amounts of cTnI and cTnT are found in human cardiac tissue [3, 24] because both cTn are expressed as an obligate 1:1 complex [2]. Once they reach the circulation, cTnI and cTnT are cleared by liver and kidneys with similar kinetics [3, 25]. Still, cTnI often reaches ten-times higher peak concentrations and decreases more quickly following MI compared to cTnT [4–6, 26]. This is due to at least two mechanisms. First, cTnI is faster cleaved than cTnT in necrotic cardiac tissue. This results in the release of cTnI fragments that can be measured with the Abbott assay. Second, most cTnT remains bound to insoluble cardiomyocyte filaments [3, 4] and its degradation likely occurs locally by phagocytes. Hence, a relatively smaller fraction of cTnT reaches the circulation [4, 27]. These issues explain the stronger association of hs-cTnI with the hs-cTn I/T ratio relative to hs-cTnT, and why type 1 MI patients, i.e. those suffering from acute myocardial necrosis, had the highest absolute values and 2-hour changes of this metric. Accordingly, the diagnostic properties of the hs-cTn I/T ratio with respect to type 1 MI appear to depend mainly on the inclusion of hs-cTnI concentrations.

Interestingly, the hs-cTn I/T ratio also distinguished type 1 from type 2 MI. The AUC was numerically higher compared to the corresponding estimates for hs-cTnI or hs-cTnT alone. This indicates that the ratio integrates information on disease processes beyond that provided by either hs-cTnI or hs-cTnT. Type 2 MI patients tend to be older compared to those with type 1 MI, and have a greater burden of chronic cardiovascular and renal illnesses [23]. Such chronic entities appear to be associated with non-necrotic cTn release mechanisms contributing to higher concentrations of circulating cTnT relative to cTnI. Support comes from studies demonstrating such cTn concentration differences in patients with stable cardiovascular or kidney disease [7–12], and from the low hs-cTn I/T ratio seen in our patients who did not have MI. The hs-cTn I/T ratio in patients with type 2 MI in contrast, was close to 1 indicating the presence of both necrotic and non-necrotic cTn release mechanisms. Along this line, our prognostic analysis indicated a stronger association of hs-cTnT with non-cardiovascular mortality as reflected by odds ratios well below 1 for the hs-cTn I/T ratio. Discrepancies in the predictive capacities of both cTn have also been observed in other studies performed in the general population [12], patients with atrial fibrillation [9] and stable coronary artery disease [7].

The results presented here suggest that the hs-cTn I/T ratio carries information that may be useful in the diagnostic work-up of chest pain patients. Considering that almost 40% of our study population had isolated elevation of hs-cTnT concentrations, this appears mainly to apply to settings where the Roche assay is used. Our findings contrast to a previous investigation reporting disappointing results in this regard [28]. That study however, considered an unselected chest pain population. The present investigation in contrast, applied a clinically more relevant approach by focusing on patients having elevated hs-cTn concentrations. This likely explains our more favorable results which corroborate with data from a smaller investigation in patients hospitalized for type 1 MI or COVID-19 infection [29]. Our findings are important since patients with an acute coronary cause of hs-cTn elevation (i.e. type 1 MI) commonly need expedited invasive assessment whereas more individualized approaches are needed in other patients with hs-cTn elevation.

Our data also suggest that the hs-cTn I/T ratio may be a clue for the clinically challenging distinction of type 2 MI from type 1 MI. Previous data have demonstrated that hs-cTn concentrations themselves only provide limited discriminative value [23, 30–32]. This could be enhanced by combinations with other cardiovascular biomarkers or advanced statistical modelling [23, 30, 31, 33], none of which currently used in routine practice.

However, even with respect to future clinical applications of the hs-cTn I/T ratio, some issues warrant consideration. First, different hs-cTn assays are rarely used in parallel at one hospital. Second, simultaneous analyses of hs-cTnT and hs-cTnI will require substantial educational efforts and should be restricted to those patients in whom differential diagnostic problems persist despite adequate initial ED assessment.

This study has some limitations. Our study population was only of moderate size and the majority of patients was enrolled several years ago. The number of patients with type 2 MI was rather small. Some caution is thus, required when interpreting our results. There were variations in the adjudication procedures between the four studies, and adjudication of type 2 MI was based on clinical judgement rather than on strictly defined criteria. This may have some bearing on reported disease prevalences among the five study cohorts, in particular regarding type 2 MI. Heterogeneity also exists between the study cohorts with respect to sex, prior manifestations of cardiovascular disease, symptom duration and diagnoses. This may have contributed to differences in hs-cTn I/T ratios, in particular between the ADAPT Brisbane cohort and the four cohorts from New Zealand. However, all multivariable analyses were adjusted for study cohort in order to minimize the potential impact of heterogeneity. We have no information on the presence of acute vs chronic myocardial injury since these entities had not been adjudicated specifically. Some patients with an evident >20% change in hs-cTn concentrations were not classified as MI with the reference contemporary cTn assay. This could have resulted in an underestimation of the true prevalence of MI and may have been different if a hs-cTn assay had been used as reference standard. We cannot exclude some incorporation bias since two studies (SPACE, EDACS) used a hs-cTn assay as reference test. It may be that calibration differences between the hs-cTn assays could have influenced our results [26]. Moreover, there is a lack of standardization between hs-cTnI assays which limits extrapolation of our findings to other assays. When interpreting the results from the multivariable analyses, one should keep in mind that the onset of symptoms is an imprecise estimate of the event of myocardial injury. We lack information on non-fatal events during 1 year of follow-up, and our prognostic data should be considered with caution due to the small number of deaths. Given the moderate sample size and limitations of exploring the validity of a model within the context it has been generated, confirmation of our findings in independent cohorts is warranted. Finally, we want to emphasize that our study is exploratory in nature, and should be regarded as hypothesis-generating.

## Conclusion

The hs-cTn I/T ratio may be useful for the early diagnosis of type 1 MI and its discrimination from type 2 MI in chest pain patients presenting with elevated hs-cTn. This probably reflects differences in release mechanisms of both cTn. Application of the hs-cTn I/T ratio may thus, facilitate early diagnostic work-up and decision-making in this clinically challenging group.

## Supporting information

S1 Appendix Adjudication procedures. (DOCX)

S1 Table Clinical characteristics and 1-year outcome per study cohort. (DOCX)

S2 Table Hs-cTn concentrations, hs-cTn I/T ratio values and their changes in patients with available data at 0 and 2 hours. (DOCX)

S1 Fig Distribution of hs-cTn concentrations in relation to type 1 MI vs no type 1 MI. A) Total cohort; B) Patients with hs-cTnT <100 ng/L and hs-cTnI <500 ng/L.
(DOCX)

S2 Fig Distribution of hs-cTn concentrations in relation to type 1 MI vs type 2 MI. A) Total cohort; B) Patients with hs-cTnT <100 ng/L and hs-cTnI <500 ng/L.
(DOCX)

S3 Fig Sensitivities and specificities of hs-cTn I/T ratio values regarding type 1 MI. (DOCX)
