# Supplementary material for: Diagnostic and prognostic performance of the ratio between high-sensitivity cardiac troponin I and troponin T in patients with chest pain
Source: PLoS One. 2022 Nov 1;17(11):e0276645. doi: 10.1371/journal.pone.0276645 (PMC9624427; doi:10.1371/journal.pone.0276645)
Supplement: S1 Appendix — (DOCX) [file pone.0276645.s001.docx]

**S1 Appendix - Adjudication procedures**

**ADAPT**

Diagnostic adjudication was performed in ADAPT by local cardiologists with access to clinical records, ECGs, cardiac troponin (cTn) results and all subsequent investigations from standard care. All adjudicators were blinded to each other's determinations and clinical site diagnoses. In cases of disagreement between the adjudicators, a consensus regarding the diagnosis of myocardial infarction (MI) was reached between two cardiologists and an emergency physician.

In a second stage, all patients with a reference cTn concentration above the 99^th^ percentile were re-adjudicated for type 2 MI. Cases were examined by a cardiologist and an emergency physician based on clinical records, cTn concentrations and the results of investigations performed up to 30 days from emergency department (ED) presentation. Underlying coronary artery disease was not required for a diagnosis of type 2 MI. In cases of disagreement, a consensus was reached between the two adjudicators.

**ADAPT-ADP, EDACS, SPACE**

The diagnosis of MI was adjudicated in the ADAPT-ADP, EDACS and SPACE studies by central committees consisting of three members, two of which being cardiologists and the other an emergency physician. All adjudicators were experienced in clinical trial adjudication, blinded to each other's determinations and clinical site diagnoses, and adjudicated index events separately. In cases of disagreement between two adjudicators in any study, differences were resolved by the third adjudicator.

In a second stage, clinical records were screened out for all patients with a reference cTn concentration above the 99^th^ percentile at any measurement instance within 24 hours from ED presentation. This included patients initially adjudicated as having MI. A single cardiologist then adjudicated these cases for type 2 MI based on the information provided in clinical records, ECGs, cTn results and results from investigations performed as part of standard care. Again, underlying coronary artery disease was not required for a diagnosis of type 2 MI.
