# Supplementary material for: Diagnostic and prognostic performance of the ratio between high-sensitivity cardiac troponin I and troponin T in patients with chest pain
Source: PLoS One. 2022 Nov 1;17(11):e0276645. doi: 10.1371/journal.pone.0276645 (PMC9624427; doi:10.1371/journal.pone.0276645)
Supplement: S1 Table — (DOCX) [file pone.0276645.s002.docx]

**S1 Table. Clinical characteristics and 1-year outcome per study cohort.**

|  | **ADAPT Christchurch (n=384)** | **ADAPT Brisbane (n=170)** | **SPACE (n=94)** | **ADAPT-RCT**  **(n=159)** | **EDACS**  **(n=81)** |
| --- | --- | --- | --- | --- | --- |
|  |  |  |  |  |  |
|  |  |  |  |  |  |
| **Demographics** |  |  |  |  |  |
| Age (years) | 73.5  (63.2-80.5) | 70.0  (59.8-80.0) | 69.5  (60.5-80.0) | 70.9  (61.9-77.3) | 66.8  (58.6-75.4) |
| Men | 249 (64.8%) | 101 (59.4%) | 74 (78.7%) | 120 (75.5%) | 63 (77.8%) |
|  |  |  |  |  |  |
| **Risk factors** |  |  |  |  |  |
| Previous smoking | 51 (13.3%) | 32 (18.8%) | 12 (12.8%) | 23 (14.5%) | 13 (16.0%) |
| Hypertension | 263 (68.5%) | 124 (72.9%) | 57 (61.3%) | 98 (61.6%) | 53 (65.4%) |
| Diabetes | 87 (22.7%) | 43 (25.3%) | 17 (18.1%) | 35 (22.0%) | 15 (18.5%) |
| Hyperlipidemia | 225 (58.6%) | 113 (66.5%) | 70 (75.3%) | 92 (57.9%) | 52 (64.2%) |
| Body mass index (kg/m^2^)* | 27.5  (24.6-31.0) | 26.6  (22.7-31.5) | 28.3  (25.0-32.6) | 27.8  (24.8-31.8) | 28.4  (25.5-31.3) |
| eGFR (mL/min/1.73m^2^)† | 61.1  (46.3-75.7) | 67.0  (43.8-83.0) | 70.2  (58.4-79.3) | n.a. | 66.7  (56.2-77.0) |
|  |  |  |  |  |  |
| **Comorbidities** |  |  |  |  |  |
| Previous MI | 148 (38.5%) | 63 (37.1%) | 44 (46.8%) | 64 (40.3%) | 29 (35.8%) |
| Previous PCI | 99 (25.0%) | 35 (20.6%) | 34 (36.2%) | 59 (37.1%) | 28 (34.6%) |
| Previous CABG | 53 (13.8%) | 32 (18.8%) | 14 (14.9%) | 23 (14.5%) | 5 (6.2%) |
| Heart failure | 69 (18.0%) | 26 (15.3%) | 9 (9.6%) | 10 (6.3%) | 4 (4.9%) |
| Previous stroke | 31 (8.1%) | 33 (19.4%) | 4 (4.3%) | 30 (18.9%) | 7 (8.6%) |
| PAD | 26 (6.8%) | 12 (7.1%) | 10 (10.6%) | 12 (7.5%) | 11 (13.6%) |
|  |  |  |  |  |  |
| **Time from onset of symptoms (hours)**‡ | 6.8 (2.9-15.4) | 4.6 (1.8-25.0) | 2.8 (1.9-6.9) | 3.8 (1.9-7.0) | 2.8 (1.5-7.5) |
|  |  |  |  |  |  |
| **Ischemic ECG** | 99 (25.8%) | 22 (12.9%) | 24 (25.5%) | 23 (14.5%) | 8 (9.9%) |
|  |  |  |  |  |  |
| **hs-cTn results** |  |  |  |  |  |
| hs-cTnI (ng/L) | 53 (17-472) | 26 (11-86) | 38 (15-245) | 35 (14-203) | 64 (22-229) |
| hs-cTnT (ng/L) | 38 (21-113) | 28 (19-53) | 30 (18-79) | 27 (19-66) | 39 (22-84) |
| hs-cTn I/T ratio | 1.67  (0.71-4.51) | 0.89  (0.44-2.32) | 1.56  (0.67-4.26) | 1.41  (0.67-3.85) | 1.64  (1.07-3.16) |
|  |  |  |  |  |  |
| **Diagnosis** |  |  |  |  |  |
| Type 1 MI | 204 (53.1%) | 39 (22.9%) | 40 (42.6%) | 78 (49.1%) | 47 (58.0%) |
| Type 2 MI | 19 (4.9%) | 31 (18.2%) | 4 (4.3%) | 0 | 2 (2.5%) |
| Myocardial injury | 161 (41.9%) | 100 (58.8%) | 50 (53.2%) | 81 (50.9%) | 32 (39.5%) |
|  |  |  |  |  |  |
| **1-year outcome**§ |  |  |  |  |  |
| All-cause mortality | 36 (9.4%) | 14 (12.1%) | 8 (8.5%) | 8 (5.0%) | 3 (3.7%) |
| • CV mortality | 25 (6.5%) | 7 (6.0%) | 5 (5.3%) | 5 (3.1%) | 3 (3.7%) |
| • Non-CV mortality | 11 (2.9%) | 7 (6.0%) | 3 (3.2%) | 3 (1.9%) | 0 |
|  |  |  |  |  |  |

*n= 821; †n=725; ‡=869; §=834.

eGFR: estimated glomerular filtration rate; MI: myocardial infarction; PCI: percutaneous coronary intervention; CABG: coronary artery bypass grafting; PAD: peripheral artery disease; cTn: cardiac troponin; MI: myocardial infarction; CV: cardiovascular.
