# Supplementary material for: Diagnostic and prognostic performance of the ratio between high-sensitivity cardiac troponin I and troponin T in patients with chest pain
Source: PLoS One. 2022 Nov 1;17(11):e0276645. doi: 10.1371/journal.pone.0276645 (PMC9624427; doi:10.1371/journal.pone.0276645)
Supplement: S2 Table — (DOCX) [file pone.0276645.s003.docx]

**S2 Table. Hs-cTn concentrations, hs-cTn I/T ratio values and their changes in patients with available data at 0 and 2 hours.**

|  | **Type 1 MI**  **(n=356)** | **Type 2 MI**  **(n=51)** | **Myocardial injury**  **(n=376)** | **Total**  **(n=783)** |
| --- | --- | --- | --- | --- |
|  |  |  |  |  |
|  |  |  |  |  |
| **0 hours** |  |  |  |  |
| hs-cTnT (ng/L) | 59 (30-151) | 29 (20-45) | 21 (16-29) | 29 (18-164) |
| hs-cTnI (ng/L) | 137 (48-715) | 29 (16-52) | 14 (8-24) | 30 (12-156) |
| hs-cTn I/T ratio | 2.60 (1.33-5.79) | 0.93 (0.68-1.77) | 0.63 (0.38-1.06) | 1.13 (0.57-2.95) |
|  |  |  |  |  |
| **2 hours** |  |  |  |  |
| hs-cTnI (ng/L) | 261 (81-1324) | 44 (27-109) | 15 (8-25) | 40 (15-258) |
| hs-cTnT (ng/L) | 79 (41-224) | 35 (25-54) | 21 (16-29) | 32 (19-85) |
| hs-cTn I/T ratio | 3.73 (1.91-6.84) | 1.18 (0.81-2.00) | 0.66 (0.39-1.10) | 1.39 (0.65-3.94) |
|  |  |  |  |  |
| **Δ 0/2 hours** |  |  |  |  |
| hs-cTnI (%) | 42.5 (8.5 to 132.3) | 10.7 (-1.3 to 128.6) | 5.1 (-4.8 to 17.3) | 13.7 (0.0 to 59.9) |
| hs-cTnT (%) | 21.2 (4.4 to 64.7) | 4.2 (-3.4 to 79.7) | -0.7 (-7.4 to 8.7) | 5.6 (-3.7 to 30.0) |
| hs-cTn I/T ratio (%) | 19.4 (3.2 to 55.0) | 10.5 (0.9 to 51.0) | 5.6 (-4.2 to 18.7) | 10.6 (-1.1 to 33.5) |
|  |  |  |  |  |

MI: myocardial infarction.

MI: myocardial infarction.
