# Supplementary material for: Diagnostic and prognostic performance of the ratio between high-sensitivity cardiac troponin I and troponin T in patients with chest pain
Source: PLoS One. 2022 Nov 1;17(11):e0276645. doi: 10.1371/journal.pone.0276645 (PMC9624427; doi:10.1371/journal.pone.0276645)
Supplement: S2 Fig — A) Total cohort; B) Patients with hs-cTnT <100 ng/L and hs-cTnI <500 ng/L. (DOCX) [file pone.0276645.s005.docx]

**S2 Figure. Distribution of hs-cTn concentrations in relation to type 1 MI vs type 2 MI. A) Total cohort; B) Patients with hs-cTnT <100 ng/L and hs-cTnI <500 ng/L.**

| **A)** | **** |
| --- | --- |
| **B)** | **** |

The dotted line represents a hs-cTn I/T ratio of 1.

MI: myocardial infarction.
