# Supplementary material for: Diagnostic and prognostic performance of the ratio between high-sensitivity cardiac troponin I and troponin T in patients with chest pain
Source: PLoS One. 2022 Nov 1;17(11):e0276645. doi: 10.1371/journal.pone.0276645 (PMC9624427; doi:10.1371/journal.pone.0276645)
Supplement: S3 Fig — (DOCX) [file pone.0276645.s006.docx]

**S3 Fig. Sensitivities and specificities of hs-cTn I/T ratio values regarding type 1 MI.**

Sensitivities (red dots) and specificities (blue dots) are presented with 95% confidence intervals. Hs-cTn I/T ratio values are dichotomized by decile boundaries.

MI: myocardial infarction.
